# Topographic imaging with automatic z-axis correction of *Brassica oleracea* var. *viridis* leaves by IR-MALDESI mass spectrometry imaging

**DOI:** 10.1007/s00216-025-05820-4

**Published:** 2025-03-15

**Authors:** Quinn Mills, Sarah M. Ashbacher, Alexandria L. Sohn, David C. Muddiman

**Affiliations:** Biomolecular Imaging Laboratory for Disease and Exposure Research (BILDER), Department of Chemistry, North Carolina State University, Raleigh, NC USA

**Keywords:** IR-MALDESI, Mass spectrometry imaging, Plant imaging, Plant metabolomics, Plant biology, *Brassica oleracea* var. *viridis*

## Abstract

Mass spectrometry (MS) is a versatile technique for elucidating the chemical composition of biological samples. Beyond analysis of crude extracts, MS can be further applied to spatially resolve compounds across the area of a sample with a technique called mass spectrometry imaging (MSI). The infrared matrix-assisted laser desorption ionization (IR-MALDESI) platform combines elements of matrix-assisted laser desorption ionization (MALDI) and electrospray ionization (ESI) to enable MSI of mammalian tissue using endogenous water in the sample as a matrix. For laser-based techniques such as IR-MALDESI, changes in topography across the sample surface cause inconsistent ablation as the sample surface moves above and below the focal plane of the laser. The localization of chemical species in plants reveals crucial information about metabolic processes as reported by Nemes and Vertes (*Anal. Chem.* 79 (21), 8098–8106, 2007) and biosynthetic pathways by Zou et al. (*Trends in Plant Science*, 2024) and can even inform selective breeding of crops as discussed by Sakurai (*Breed Sci* 72 (1), 56–65, 2022); however, leaf topography raises a unique challenge. Features such as veins and trichomes exhibit unique topography, but flattening risks delocalization of analytes and activation of unwanted signaling pathways, and transferring metabolites to a membrane for indirect analysis may incur delocalization and limit metabolomic coverage. To overcome these challenges, a chromatic confocal sensor probe (CA probe) was incorporated for IR-MALDESI-MSI of sections of a collard (*Brassica oleracea* var. *viridis*) leaf. The CA probe measures the height at all points of the sample, and automatic z-axis corrections (AzC) are generated from height differences to continuously raise and lower the stage. These stage height corrections keep the sample surface in focus of the laser for the duration of analysis. This method has been applied to relatively homogenous samples, but has not yet been characterized on heterogeneous leaf tissue with considerable topography. Herein, data quality is compared between MSI analyses with and without AzC applied, focusing on the localization of analytes known to be concentrated in different layers of collard leaves.

**Graphical Abstract:**

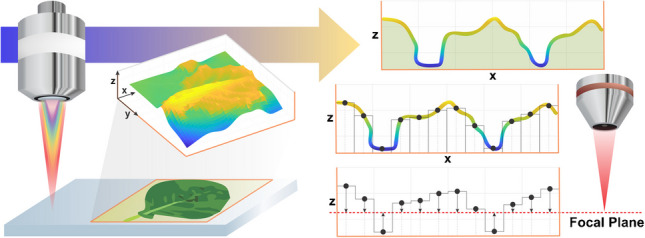

**Supplementary Information:**

The online version contains supplementary material available at 10.1007/s00216-025-05820-4.

## Introduction

Mass spectrometry (MS) is a robust and widely applied analytical technique to identify the chemical components of a sample. For research in plant biology, MS analysis has become an essential tool for metabolomics research, offering insight into metabolic and biosynthetic pathways [1], stress signaling responses [2], symbiotic relationships [3], and phenotypic differences [4]. Research in these areas is crucial for advancing diverse fields including agriculture [5–7], food science [8–10], and natural products discovery [11–13]. Identification of structurally similar metabolites can help piece together biosynthetic pathways to guide chemical synthesis of natural products, leading to advancements in drug discovery pipelines.

Currently, hyphenated techniques (e.g., GC-MS, LC-MS, and HPLC-MS) still dominate plant biology research. While the biological data generated from these analyses is crucial, these methods often require the preparation of a liquid extract from plant tissue. Extraction can be costly and time-consuming, requires solvents, and often limits metabolomic coverage by favoring certain molecular classes [14]. Beyond these limitations, extraction eliminates another critical but largely unexplored piece of the botanical puzzle: the spatial localization of plant metabolites.

By collecting mass spectra at an array of discrete locations across a sample, MSI allows researchers not only to putatively identify chemical species, but to examine their spatial localization across a sample. Each spectrum represents the abundance and *m*/*z* data from one voxel sampled, which then corresponds to a pixel in the ion image. Ion images are generated as heat maps of the abundance of a chosen *m*/*z*, allowing relative quantification of analytes. The resulting localization information can deepen understanding of plant metabolomics by demonstrating colocalization of metabolites and their localization to different structures in the tissue. While hyphenated MS analysis is common in plant metabolomics research, adaptation of MSI methods has been relatively slow, owing in part to the unique challenges presented by leaf tissue.

Plants feature a complex and variable morphology, and leaves broadly consist of four distinct tissues: the cuticle, epidermis, palisade mesophyll, and spongy mesophyll. Each of these layers contains accumulated secondary metabolites responsible for important functions in plants. The outermost layer is the cuticle, a hydrophobic layer of waxes and lipids that primarily controls the exchange of water with the environment [15]. Below the cuticle is the epidermis which further protects the plant from biotic and abiotic stress with thickened cell walls and a high concentration of bioactive secondary metabolites [16]. The innermost layer is the mesophyll which can be further divided into the palisade mesophyll and spongy mesophyll. Palisade mesophyll cells are elongated and tightly packed, and their high numbers of chloroplasts are responsible for the majority of photosynthesis occurring in the plant. The loosely packed cells of the spongy mesophyll supplement photosynthesis but are mainly responsible for gas regulation [17]. At present, challenges in MSI of plants result largely from obstruction by the cuticle, high water content, and sample topography. IR-MALDESI bypasses the cuticle with a 2.97-μm laser that incorporates the high water content by exciting the O-H stretching bands of endogenous water within the leaf [18]; however, it is generally restricted to analysis of flat tissues.

Traditional laser-based ionization methods require a flat tissue sample for consistent ablation throughout MSI analysis. The laser is focused at a particular height, so differences in topography cause the surface of the sample to move out of the focal plane as the stage moves beneath the laser. As the sample surface moves away from the focal plane, the diameter of the ablation spot increases and the depth of ablation decreases. Leaves demonstrate an uneven topography due to their comprehensive morphology as well as individual biological features (e.g., veins), so sample preparation methods must be incorporated to produce flat samples. Animal tissues are typically cryosectioned for IR-MALDESI MSI analysis [19], and although leaves may be cryosectioned for MALDI, LDI, or LA analysis of cross-sectioned tissue [20], analysis of the adaxial and abaxial surfaces presents complications. Alternative methods have included manually flattening leaves [21] and indirect analysis of a membrane [22], Teflon surface [23], TLC plate [24], or paper imprinted with metabolites [25], but all have limitations. A common application of MSI analysis of leaves is the interrogation of stress signaling pathways, but these pathways may be activated inadvertently when leaves are flattened. Flattening, matrix application, and membrane transfer can also result in delocalization of analytes, and membrane transfer generally favors certain classes of metabolites such as lipids in the leaf cuticle. To maintain sample integrity and minimize sample preparation, a previously established method for topographic IR-MALDESI-MSI using automatic z-axis corrections (AzC) generated by a CA probe [26] was applied for ambient MSI analysis of fresh *Brassica oleracea* var. *viridis* leaf sections.

Several platform-specific methods have been developed to correct for topography during MSI analysis which detect and correct for the height of the sample during analysis. Uneven surfaces on a strawberry have been imaged successfully by liquid microjunction-surface sampling probe mass spectrometry using a conductance feedback loop and a 3D printer platform, but the lateral resolution was limited to 1 mm [27]. A SIMS-TOF topographic MSI platform was developed for analysis of *Arabidopsis* roots, but SIMS is limited to elemental analysis [28]. A MALDI autofocusing technique was applied for topographic MSI of a daisy petal and clover leaf, but required a sprayed matrix [29]. The platform-specific IR-MALDESI-MSI AzC method has successfully analyzed relatively homogenous samples (e.g., liver tissue, burn paper, pill) but has not yet been challenged with the substantial topography and distinctly heterogeneous, layered tissue of a leaf. The CA probe allows analysis over a height difference of 2 mm, and IR-MALDESI allows matrix-free analysis of metabolites with a spot size of ~150 µm when using optics optimized for leaf tissue. [30]

## Experimental methods

**Fig. 1:**
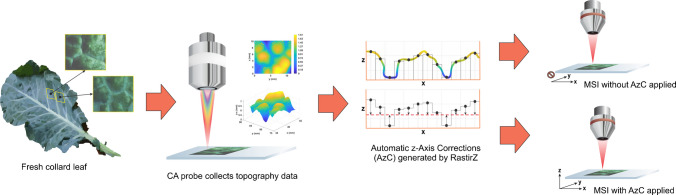
Summary of experimental workflow. From left to right: two biologically similar sections were excised from a fresh collard leaf, topography data was collected using the CA probe, AzC data were generated, and MSI data was acquired. This process was repeated first with AzC applied during MSI analysis, then again without AzC

Two leaf sections of similar size and topography were excised from nearby locations on a mature collard leaf. Topographic and MS data were collected with AzC applied for the first section and without AzC applied for the second section. For each section, topographic data were collected using the RastirZ software which automatically generated AzC data. After these data were collected, the samples were analyzed according to their respective methods. This experimental workflow is summarized in Fig. 1.

### Materials

The LC-MS grade acetonitrile (ACN), water, and formic acid (FA) used for electrospray solvent were all purchased from Fisher Scientific (Nazareth, PA, USA), as well as plain microscope glass slides (1 mm). Nitrogen gas used to dry samples was purchased from Arc Gases (Raleigh, NC, USA). Masking tape used to mount samples was purchased from Amazon (Seattle, WA, USA). Burn paper for focusing the laser was purchased from ZAP-IT (Barrington, NJ, USA). The optical train consisted of a −75 mm plano-concave beam expander (LC5401-E), +250 mm plano-convex lens (LA5255), and Schwarzschild-like reflective objective (LMM15X-P01), all purchased from Thorlabs (Newton, NJ, USA). The CA probe system consisted of an optical sensor controller and 2000-μm chromatic sensing head (Precitec GmbH & Co. KG, Gaggenau, Germany). Optical images of ROIs before analysis were collected with an in-source camera, and microscopic optical images of the ROIs were collected with the camera mounted on a laser microdissection system (LMD 7000, Leica, Wetzlar, Germany).

### Sample preparation

A whole collard plant with both mature and immature leaves was acquired locally (Raleigh, NC, USA) and refrigerated for 1 day at 2°C. Prior to collection of tissue sections, a mature leaf was removed, rinsed with ultrapure water (Purelab Flex, Veolia, High Wycombe, UK), and gently dried with nitrogen gas. To reduce biological variation, two sections were excised from adjacent locations on the margin of a mature collard leaf. Each section was between two secondary veins, such that one was directly below the other. Sections were located in a relatively uniform area, but tertiary veins supplied the necessary topography. Scissors and tweezers were wiped with methanol before excising and mounting the sections. Samples were fixed to plain glass slides with masking tape and analyzed at ambient temperature and humidity with no external matrix applied. Both samples were measured prior to analysis at 1.8 mm.

### IR-MALDESI MSI analysis and AzC

Samples were analyzed on the NextGen IR-MALDESI source [31] at atmospheric pressure, and ambient temperature and humidity. The emitter tip was aligned coaxial to the sample inlet, and the sample stage was positioned orthogonal to the emitter and inlet. The electrospray solvent was prepared as a 50% (v/v) ACN solution modified with 0.2% (v/v) FA, and a high voltage was applied to the emitter to form an electrospray plume. A 2.97-μm mid-IR laser (JGM Associates, Inc., Burlington, MA) was coupled to an optical train incorporating a Schwarzschild-like reflective objective. This optical train was previously characterized for the IR-MALDESI system [32], and recently optimized for leaf analysis [30]. With a low energy transmission to achieve laser fluence at 50 μm, the laser was tuned to deliver approximately 0.55 mJ per burst.

The source was coupled to an Orbitrap Exploris 240 mass spectrometer (Thermo Fisher Scientific, Bremen, Germany). Mass spectra were collected in positive ionization mode over a range of *m*/*z* 100–1000, with the following instrument settings: small molecule mode, centroid data, 70% S-lens RF-level, 240,000_FWHM_ resolving power at *m*/*z* 200, EASY-IC enabled, and 15 ms ion injection time with automatic gain control (AGC) disabled. Routine mass calibration with Flexmix (Pierce Biotechnology, Rockford, IL) was performed daily prior to analysis.

The method established by Xi et. al [26]. was applied in order to analyze non-flat samples by IR-MALDESI-MSI. In this method, the CA probe first scans the sample surface and measures its topography. The probe functions by dispersing white light into its component wavelengths which are aimed at the sample. The light can only converge and be reflected back into the sensor at a certain wavelength, which corresponds to a particular height. A spectrophotometer determines the wavelength of the reflected light which the instrument then uses to calculate the height of the sample stage. The RastirZ software records the height at each point of the ROI and uses these data to generate a series of automatic z-axis corrections. Each of these corrections instructs the stage to move up or down an equal but opposite distance on the z-axis to return the sample surface to the height of the focal plane, maintaining uniform ablation regardless of sample topography.

The average scan rate for acquisition of topographic data was 4.5 scans/s, and the average scan rate for acquisition of MSI data was 1.73 scans/s. Data were collected with a lateral resolution of 200 µm to prevent oversampling. The CA probe distance tolerance was increased from 10 to 20 µm, so z-axis measurements were accurate to ±20 µm. The increased tolerance increased the scan rate, ideally reducing excessive water loss or metabolomic changes during lengthy analyses.

### Data processing and analysis

Data was collected as ThermoFisher .RAW files, which were subsequently converted to .mzML with MSConvert [33] and then to .imzML with imzMLConverter [34]. A list of metabolites previously discovered in any variety of *B. oleracea* was exported from the KNApSAcK database [35], and corresponding *m*/*z* values were generated for [M+H]^+^ ions. The .imzML files were uploaded to MSiReader v2.85, and ion images were generated for each *m*/*z* using the SSIM function [36, 37]. Topographic plots were generated in Matlab with the open-source code specified in Supplemental Materials. Boxplots were generated in RStudio using abundance data exported from MSiReader and the output of z-axis coordinates generated by RastirZ, then recolored in CorelDraw.

## Results and discussion

**Fig. 2:**
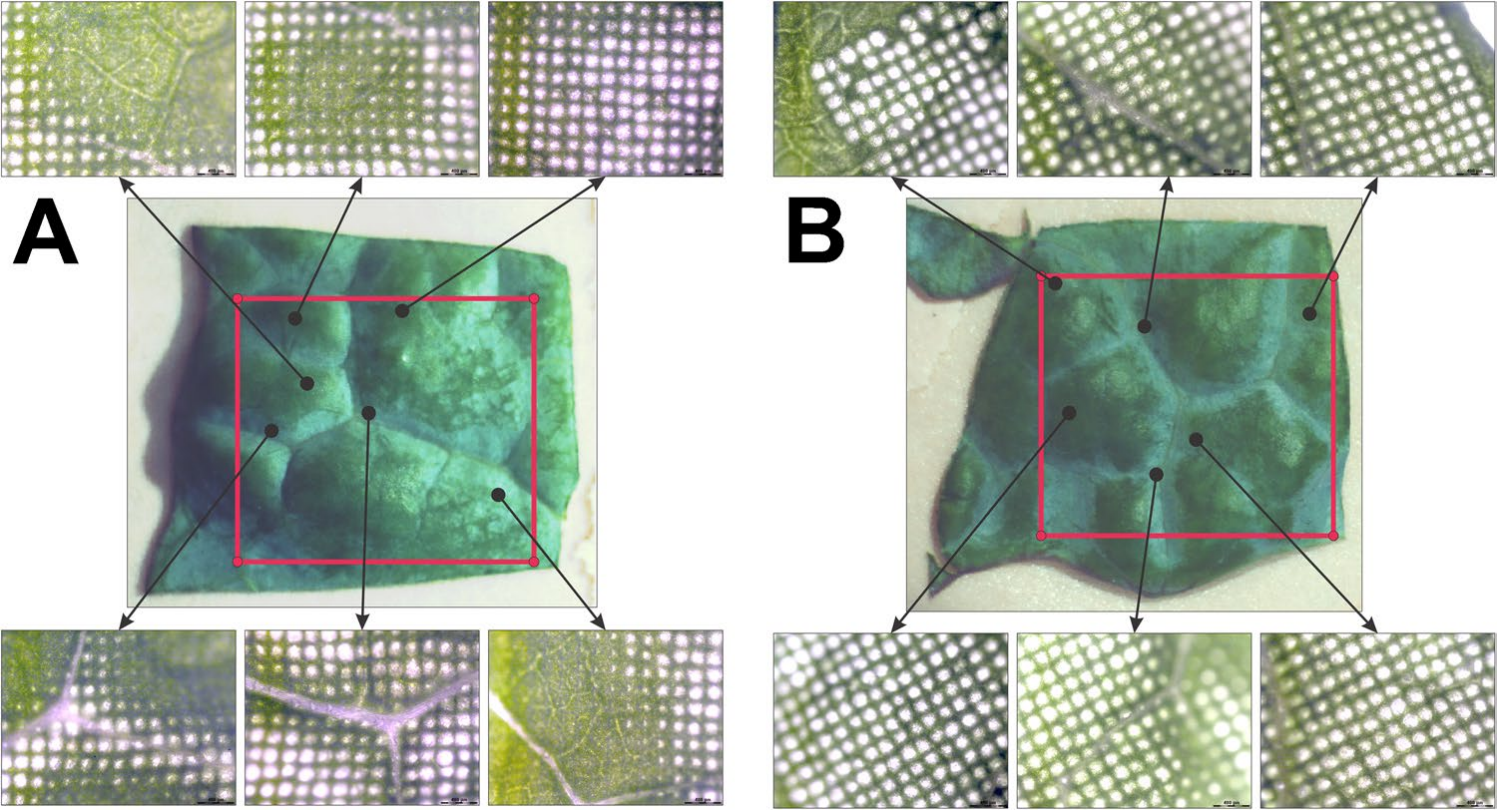
Optical images of leaf sections analyzed without AzC applied (**A**) and with AzC applied (**B**). The center images were captured with the in-source camera before analysis, and the ROIs are outlined in red. The Leica images were captured after MSI analysis, and are linked to their approximate location on the ROI indicated by the black arrows. Note that only areas of deep ablation are visible in the Leica images. Additional images are included in Supplemental Information to demonstrate areas of shallow ablation around the edges of ablation spots only visible at a higher zoom

The Leica images of the leaf section analyzed without AzC applied, shown in Fig. 2A, demonstrate a clear relationship between ablation spot shape and sample topography. The laser was focused approximately halfway between the maximum and minimum height of the sample surface. The visible diameter of the ablation spot decreases as the surface height moves away from the height of the focal plane, and ablation spots disappear entirely at extreme heights. With AzC applied, this relationship is no longer observed; the spot shape is relatively uniform and independent of sample topography. The missing ablation spots in the top left image of Fig. 2B are artifacts of CA probe measurement errors caused by steep height changes or interference. When AzC data are generated from these incorrect measurements, RastirZ erroneously moves the stage out focus. Although missing spots are not ideal, these errors are both uncommon and random; thus, they do not systematically impact the biological accuracy of the resulting data. In contrast, the differences in the volume and depth of ablation when AzC are not applied follow a clear pattern according to the topography, which systematically biases the resulting MSI data, potentially leading to biologically inaccurate conclusions.

To explore the effect on MSI data quality, abundance and colocalization patterns were evaluated for ion images of all *m*/*z* values generated from the KNApSAcK database search. A total of 12 ions were selected for further analysis. Four metabolites each were chosen to represent three classes: fatty acids (FA), amino acids (AA), and metabolites related to lignin biosynthesis (LM). Each class demonstrates a unique but consistent pattern of tissue localization. Octocosanal and 15-nonacosanone were chosen as fatty acids since their presence in the cuticle is well documented [38–40]. Two additional fatty acids, palmitic acid and methyl heptadecanoate, were chosen due to their high abundance and presence in leaf cuticle [41, 42]. Four proteinogenic amino acids, l-proline, l-valine, l-leucine, and l-phenylalanine, were chosen due to their tendency to accumulate in the epidermis [43]. They are also active in stress signaling, and originate in the mesophyll. The final four metabolites, 4-hydroxybenzoic acid, p-coumaric acid, shikimic acid, and caffeic acid, were chosen in relation to the shikimic acid pathway. Shikimic acid is the end product of the shikimic acid pathway, and is involved in the biosynthesis of lignin along with p-coumaric acid and caffeic acid, both of which can also be produced in response to abiotic stresses (e.g*.*, salinity, cold, and heat) [44]. 4-Hydroxybenzoic acid is a phenolic precursor for many metabolites which may be biosynthesized from p-coumaric acid [45]. Biosynthesis of these metabolites all begins with photosynthesis products generated in the mesophyll [46]. A summary of the chosen analytes and their corresponding major tissue layers is provided in Table 1.

**Table 1:**
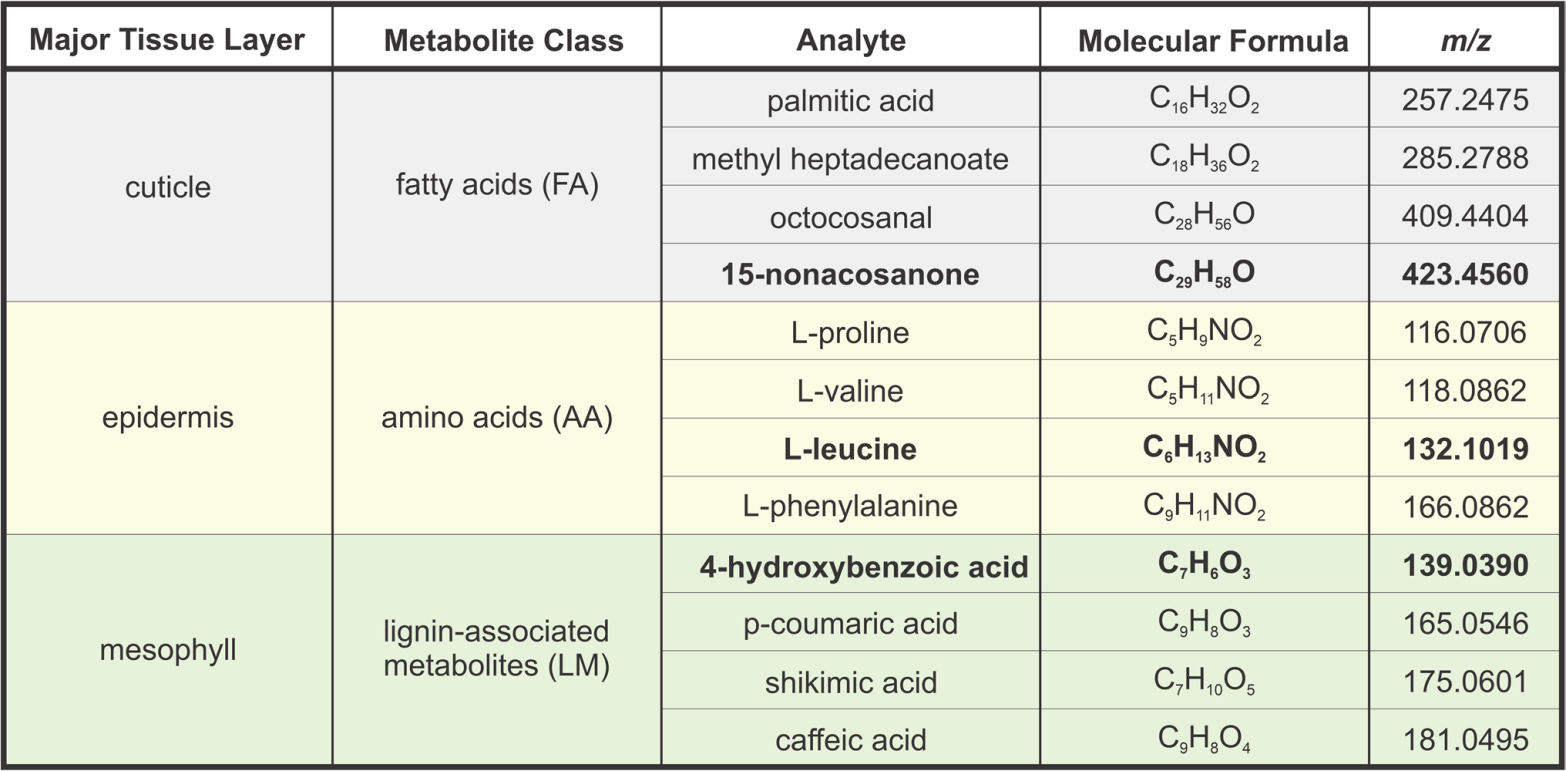
Analytes evaluated. The bolded metabolites are used to represent each class of metabolites

**Fig. 3:**
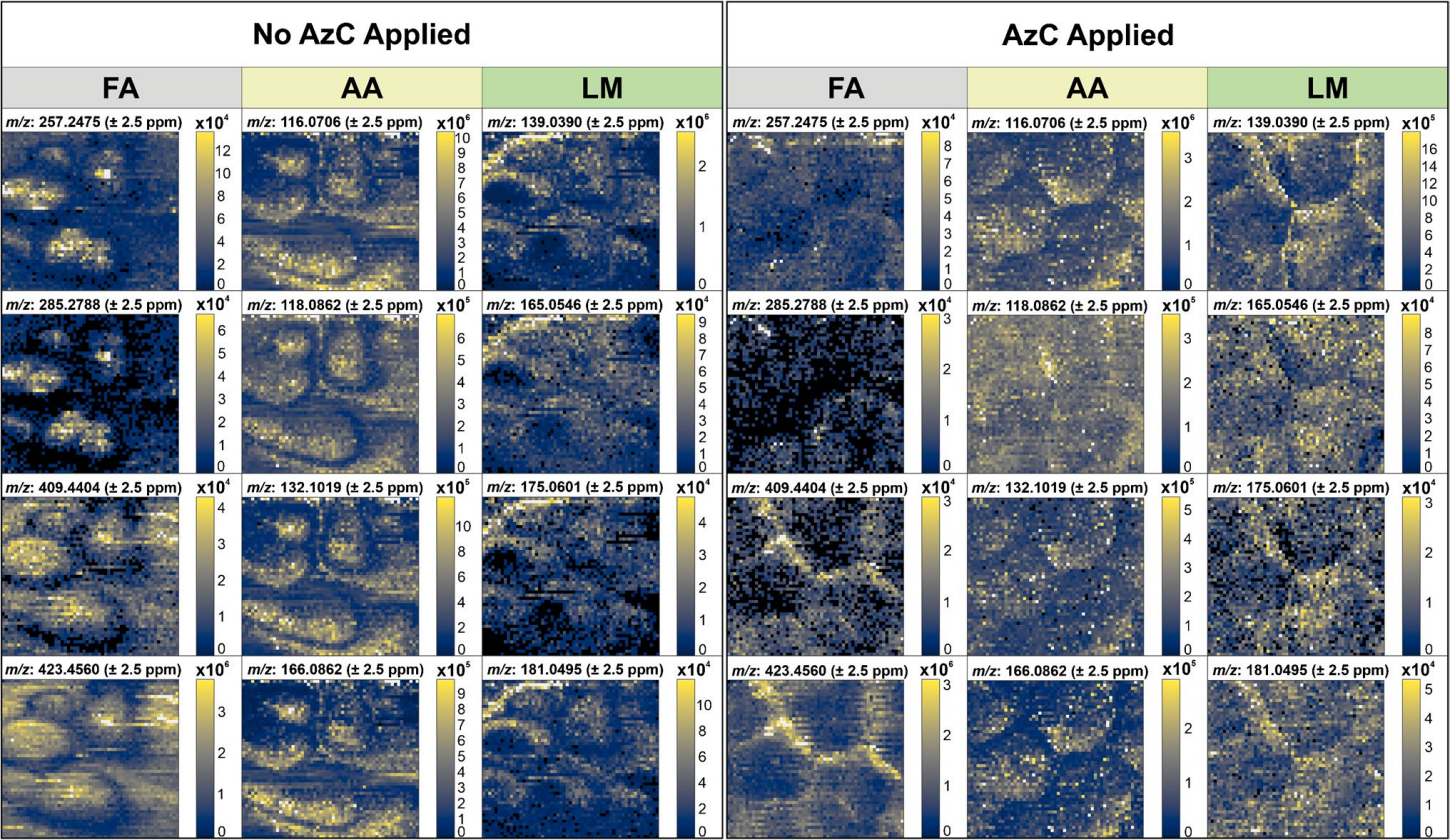
Ion images of the chosen FAs, AAs, and LMs for datasets collected with and without AzC applied. For each dataset, ion images are compiled as a column of four fatty acids on the left, a column of amino acids in the middle, and a column of lignin-associated metabolites on the right. Images collected without AzC are shown on the left, and those collected with AzC are shown on the right. The *m/z* values for all images are reported to ±2.5 ppm, and are listed in order of increasing *m*/z. From top to bottom, the FAs are *m*/*z* 257.2475, 285.2788, 409.4404, and 423.4560. From top to bottom, the AAs are *m*/*z* 116.0706, 118.0862, 132.1019, and 166.0862. From top to bottom, the LMs are *m*/*z* 139.0390, 165.0546, 175.0601, and 181.0495. All data were collected as an ROI of 2907 total scans at a resolving power of 240,000_FWHM_ at *m*/*z* 200 with a step size of 200 μm. The remaining relevant SMART parameters [47] for data collection without AzC are as follows: highly variable spot size up to approximately 130 × 130 μm of visibly ablated tissue; 37 minutes of collection time. With AzC, the parameters are as follows: spot size of approximately 130 × 130 μm; 41 minutes of collection time

The resulting ion images, portrayed in Fig. 3, yielded unexpected results. While little to no ablation was visible at high and low surface heights without AzC, the ion images show high abundances of analytes in these areas that did not appear to be sampled. Additionally, the localization patterns of the FAs and LMs appear inverted between the two datasets when compared to the biological features of their respective ROIs. Without AzC applied, FAs appear localized to areas of maximum and minimum surface height between veins, and LMs appear localized to areas of medium height between veins. With AzC applied, FAs appear localized to the veins and surrounding areas, and LMs are localized to the veins and otherwise relatively evenly distributed. While some biological variability is expected, a completely inverted localization pattern is surprising, and the Leica images suggest that this is an artifact of inconsistent ablation due to the sample topography.

Interpretation of the ion images of the representative SA, 4-hydroxybenzoic acid, is complicated by the fact that it is an isomer of salicylic acid, which is a well-documented stress signaling hormone [48]. Since the two sections were analyzed sequentially, the plant had more time to initiate a stress signaling response between analyses, which may have caused the transport or accumulation of salicylic acid to different areas prior to acquisition of MSI data. Although this could cause differences in the distribution of analytes between the two sections sampled, the colocalization of this LM with the other LMs suggests that it is more related to lignin production. This process depends more on overall plant growth and long-term stress responses [49], and variation in these factors was reduced by selecting biologically similar sections close together on the same leaf.

**Fig. 4:**
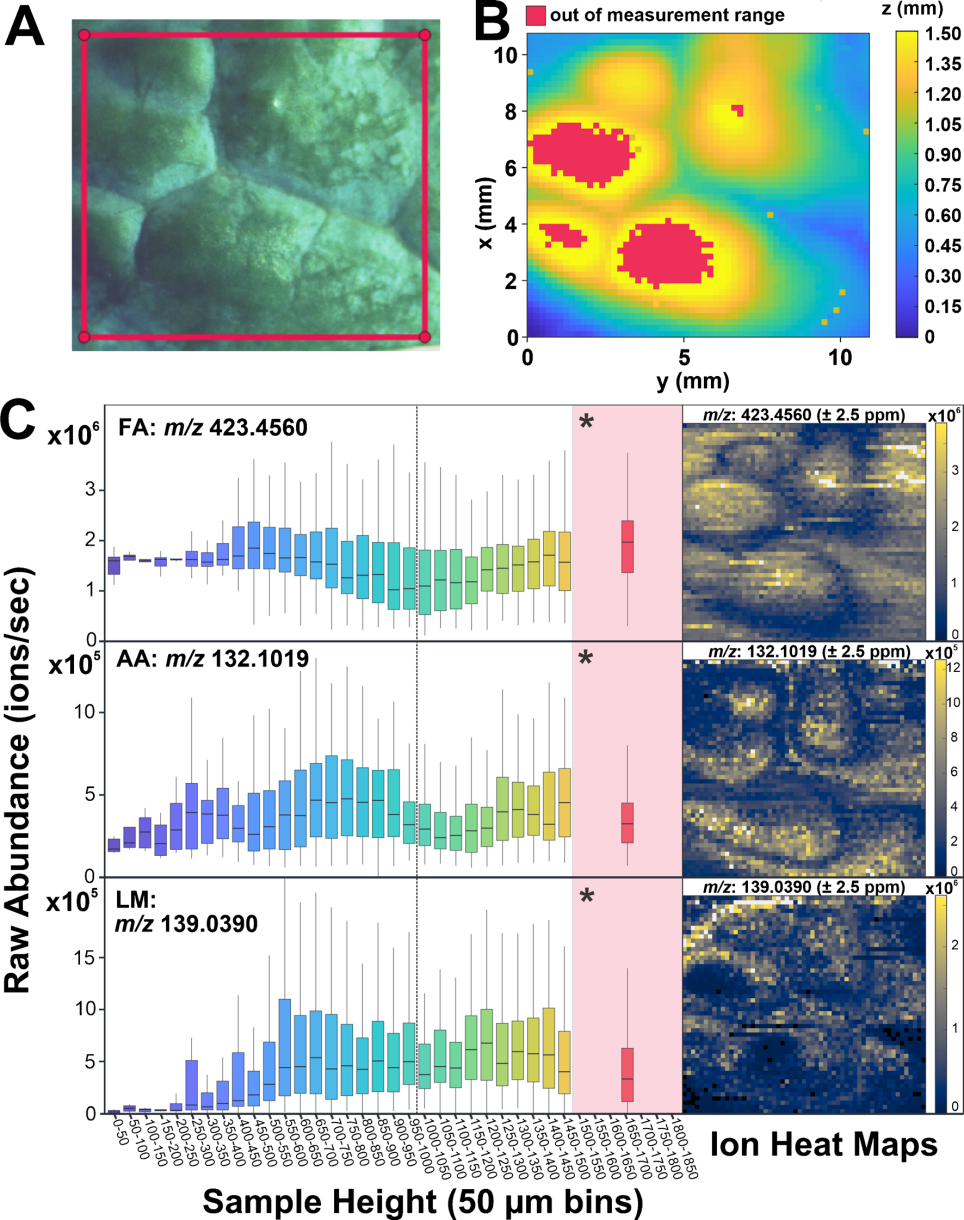
Comparing optical images, topography, and ion heat maps for leaves analyzed without AzC. (**A**) An optical image of the ROI (outlined in red) shows biological features of the leaf including veins and visibly denser areas of cuticular wax prior to analysis. (**B**) A topographic plot of the ROI maps the surface height of the sample prior to analysis. (**C**) On the right are ion heat maps of the [M+H]^+^ ion for an example FA (15-nonacosanone, *m*/*z* 423.4560), AA (l-leucine, *m*/*z* 132.1019), and LM (4-hydroxybenzoic acid, *m*/*z* 139.0390). All ion heat maps were generated with a mass measurement accuracy tolerance of ±2.5 ppm. To the left of each heat map is a corresponding boxplot of raw abundance across 50-μm bins of sample height. Due to the working principle of the probe, some of the maximum heights were outside of its working distance and had to be binned together for further data analysis. These spots are marked in red on the topographic plot. Since the leaf sections were measured with calipers before analysis and both had a total height difference of ~1800 μm, abundance data from all out-of-range heights were added to one 350-μm bin from 1500 to 1850 μm. This is highlighted in red and denoted with an asterisk

To explore the reason for this unexpected distribution of analytes, ion images of FAs, AAs, and LMs were compared directly to optical images and topographic plots of their corresponding ROIs, shown in Fig. 4. In the dataset collected without AzC, the biological features (*e.g.*, veins, whitish areas of crystallized cuticular wax [38] around the veins) in the optical image of the ROI (Fig. 4A) do not align perfectly with high and low areas of the topographic plot (Fig. 4B). For example, although the veins generally follow areas of low surface height, the bottom left corner shows veins that are elevated in the topographic plot compared to the others. The localization of analytes in the ion images (Fig. 4C) appears to be influenced more by the sample’s topography than biological features. The clearest example is the representative FA, 15-nonacosanone. This FA has been documented in *B. oleracea* [39] and is known to be a predominant component of the cuticular wax in *Arabidopsis thaliana*. [40] As a very long-chain fatty acid derivative, it only accumulates in the cuticular wax; therefore, the highest abundance should be found in the visibly thickened areas of cuticular wax around the veins. Instead, it appears to be more abundant everywhere else. Note that the veins and visible cuticle in the optical image are not as round as the bands in the ion image. The rounded edges of the low-abundance bands correspond to the rounded mid-height bands in the topographic plot more than the sharp outlines of the veins in the optical image. This is particularly clear in the bottom half of the image, where a band of low abundance corresponds to a band of medium height in the topographic image despite a lack of veins in this area. Likewise, the AA ion image includes rounded bands of low abundance that approximately correspond to bands of low, high, and medium sample height in the topography plot more than veins or concentrated cuticular wax in the optical image. The LM ion image includes areas of low abundance at the minimum and maximum topographic heights, irrespective of the location of veins and wax in the optical image.

While the differences in abundance between bins of sample surface height of Fig. 4C are not statistically significant, there are visible trends for different metabolite classes. For the FA, the abundance drops around the height of the focal plane, but is highest at the minimum and maximum topographic heights. A similar pattern is visible for the AA, but abundance decreases at minimum and maximum topographic heights, and the lower abundance area between them is centered slightly above the focal plane. This shift in height is likely due to the true localization of the AA in the sample. The trend in the LM is further exaggerated with a sharp decrease in abundance at the minimum and maximum topographic heights and an increase closer to the focal plane.

**Fig. 5:**
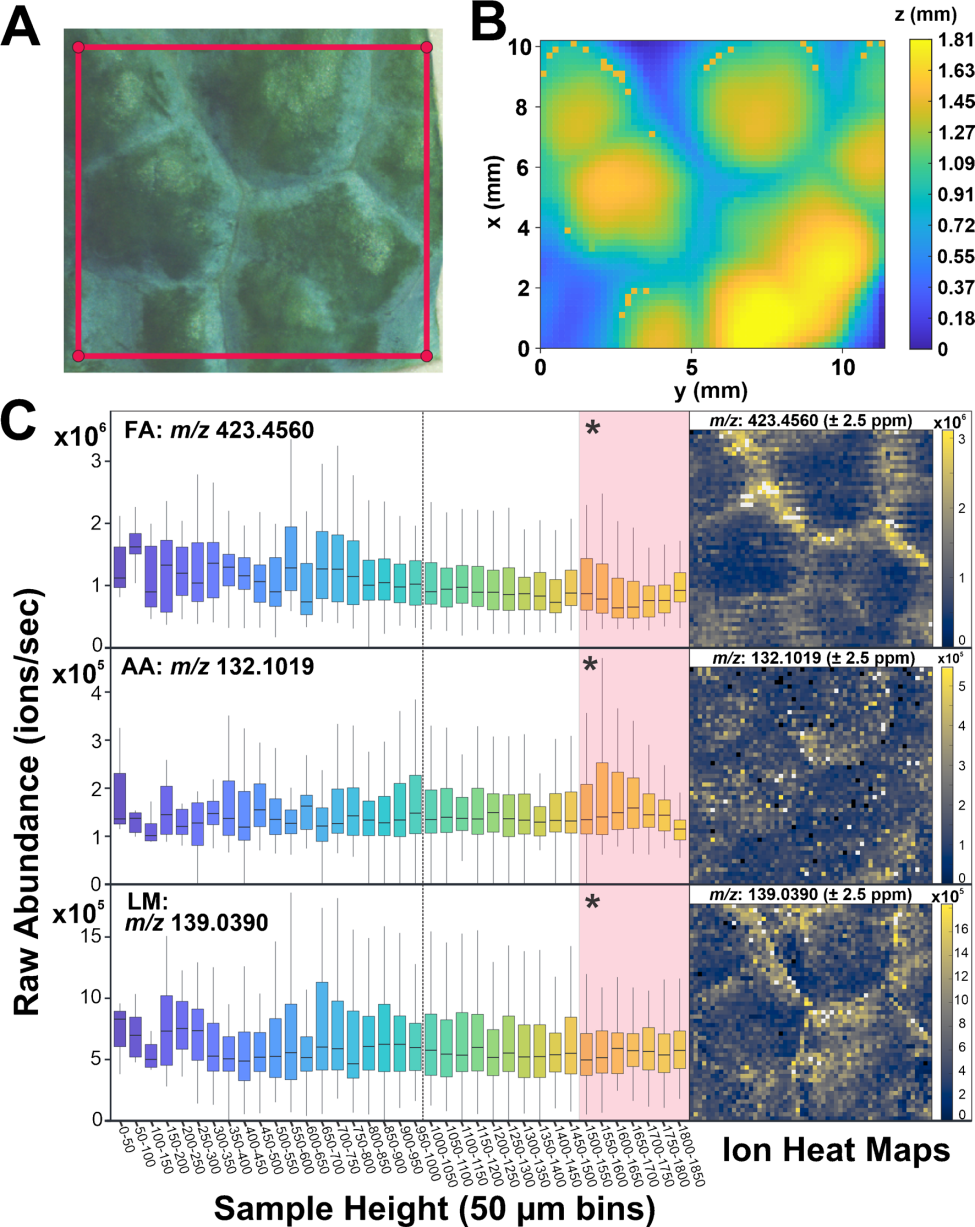
Comparing optical images, topography, and ion heat maps for leaves analyzed with AzC applied. (**A**) An optical image of the ROI (outlined in red) shows biological features of the leaf including veins and visibly denser areas of the cuticle prior to analysis. (**B**) A topographic plot of the ROI maps the surface height of the leaf section prior to analysis. (**C**) On the right are ion images of the [M+H]^+^ ion for an example FA (15-nonacosanone, *m*/*z* 423.4560), AA (l-leucine, *m*/*z* 132.1019), and LM (4-hydroxybenzoic acid, *m*/*z* 139.0390). All ion images were generated with a mass measurement accuracy tolerance of ±2.5 ppm. To the left of each ion image is a corresponding boxplot of raw abundance across 50-μm bins of sample height. All measurements in this sample were recorded within the working distance of the probe and are binned as such, but the height interval from 1500 to 1850 μm is highlighted in red for comparison with the combined bin in the other dataset

In contrast to Fig. 4, the localization patterns for the dataset sampled with AzC in Fig. 5 appear to reflect the biological features in the optical image of the ROI (Fig. 5A) more than the sample topography (Fig. 5B). In the representative ion images (Fig. 5C), localization of the FA aligns with the visibly thickened areas of cuticle around the veins in the optical image as expected. The AA appears localized to the areas between veins, regardless of height, and the LM seems particularly abundant along the veins and in certain areas between them. The localization of the LM to the veins is pronounced and follows the sharper lines of the veins rather than the rounded bands of the topography. Unlike the boxplots in Fig. 4C, the boxplots in Fig. 5C demonstrate no distinct trend in the abundance of any analytes at different topographic heights. For example, in the ion image of the FA in Fig. 5C, there is a band of high abundance transecting the image from the top-left to middle-right. This band aligns with the white area surrounding the vein in the optical image, but according to the topographic map, this band gradually covers a total height difference of approximately 1 mm. While abundance fluctuates across bins of height, there is no clear distribution around the height of the focal plane, which further supports that the localization patterns captured in the ion images reflect biology rather than topography.

**Fig. 6:**
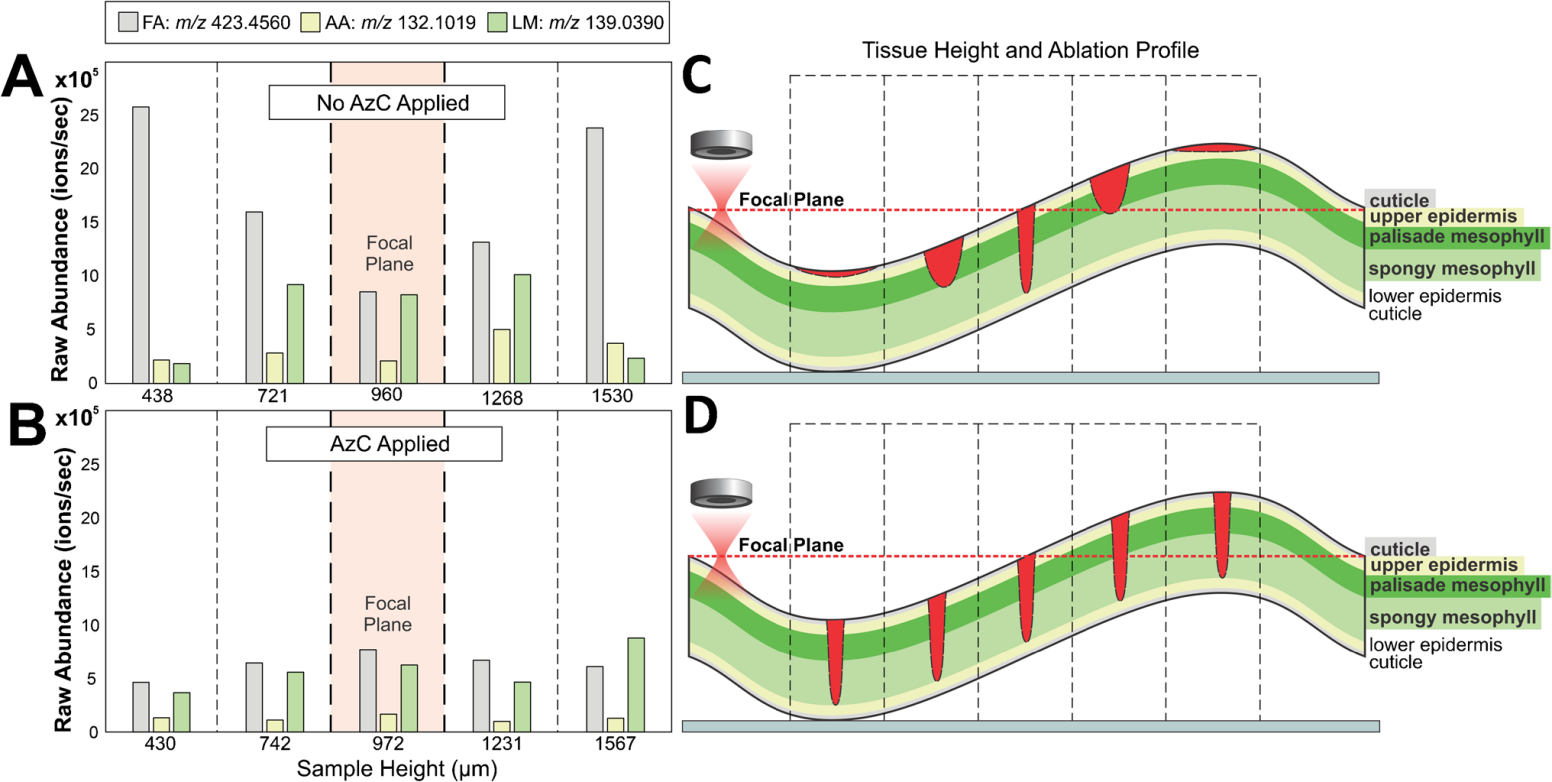
Abundance of an example FA (*m*/*z* 423.4560), AA (*m*/*z* 132.1019), and LM (*m*/*z* 139.0390) for five example ablation spots sampled without AzC (**A**) and with AzC (**B**), alongside proposed explanations for the difference in abundance trends without AzC (**C**) and with AzC (**D**). The example spots were chosen at five different sample surface heights: approximately the height of the focal plane (960 μm and 972 μm), below the focal plane (721 μm and 742 μm), farther below the focal plane (438 μm and 430 μm), above the focal plane (1268 μm and 1231 μm), and farther above the focal plane (1530 μm and 1657 μm). Spots were chosen such that the heights are comparable between the two datasets, and the height range was defined to exclude interpolated data

Although the data from the section analyzed without AzC appears to show clear localization patterns and high ion abundance, Leica images of the ablation spots suggest that these imaging data are inaccurate and influenced by the topography of the sample. A mechanism is proposed in Fig. 6 to explain why some analytes were measured with high abundance in areas that appeared unsampled, and why the localization of some analytes appeared inverted between the datasets collected with and without AzC. These unexpected data may have resulted not only from the change in laser spot diameter and depth of ablation farther from the laser’s focal plane, but also from the localization of different analytes to layers of leaf tissue at different depths. Without AzC (Fig. 6A), the FAs appeared most affected by sample topography; the highest abundance of FAs was detected at the maximum and minimum heights. No ablation was visible in the Leica images in these areas of the tissue, but the images cannot capture shallow ablation. For this method, the depth of ablation decreases and the spot diameter increases as the sample moves farther from the focal plane of the laser, resulting in a larger volume of surface tissue ablated (Fig. 6C). Following this pattern, analytes localized to the cuticle should have a maximum ablation volume at minimum and maximum topographic heights. As the depth of the tissue layer increases, the height where the largest volume of that tissue is ablated should approach the height of the focal plane. In contrast, the abundance of the FA, AA, and LM are similar across surface heights when AzC are applied (Fig. 6B). As demonstrated in (Fig. 6D), the ablation volume should be consistent across all tissue layers for this method. The lower layers are always sampled, but only a small amount of the cuticle and epidermis are sampled regardless of topographic height.

## Conclusion

The CA probe successfully captured the topography of different sections of collard leaves and translated those measurements into AzC data. When those data were applied, the depth and diameter of the laser spots were relatively uniform, independent of height variations across the sample. Conversely, the spot shape depended heavily on the surface height of the sample without AzC applied; Leica images after analysis showed that the diameter of deeply ablated tissue decreased for spots above and below the focal plane. The effects of topography on the volume of different tissue layers ablated influenced localization patterns in the resulting ion images. Localization patterns of the FAs in the cuticle even appeared to be inverted without AzC. These inaccuracies, which are difficult to discern from biologically accurate MSI data since they reflect topographic patterns and thus appear as well-differentiated regions, could lead to incorrect biological conclusions. In the future, this AzC method may be applied for topographic MSI of other agriculturally, medically, or biologically important plants.

## Supplementary Information

Below is the link to the electronic supplementary material.

Supplementary file1 (PDF 2.36 MB)

Supplementary file2 (XLSX 38 KB)
