# Supplementary material for: Topographic imaging with automatic z-axis correction of Brassica oleracea var. viridis leaves by IR-MALDESI mass spectrometry imaging
Source: Anal Bioanal Chem. 2025 Mar 15;417(11):2321–32. doi: 10.1007/s00216-025-05820-4 (PMC11996940; doi:10.1007/s00216-025-05820-4)
Supplement: Supplementary file 1 — Supplementary file1 (PDF 2.36 MB) [file 216_2025_5820_MOESM1_ESM.pdf]

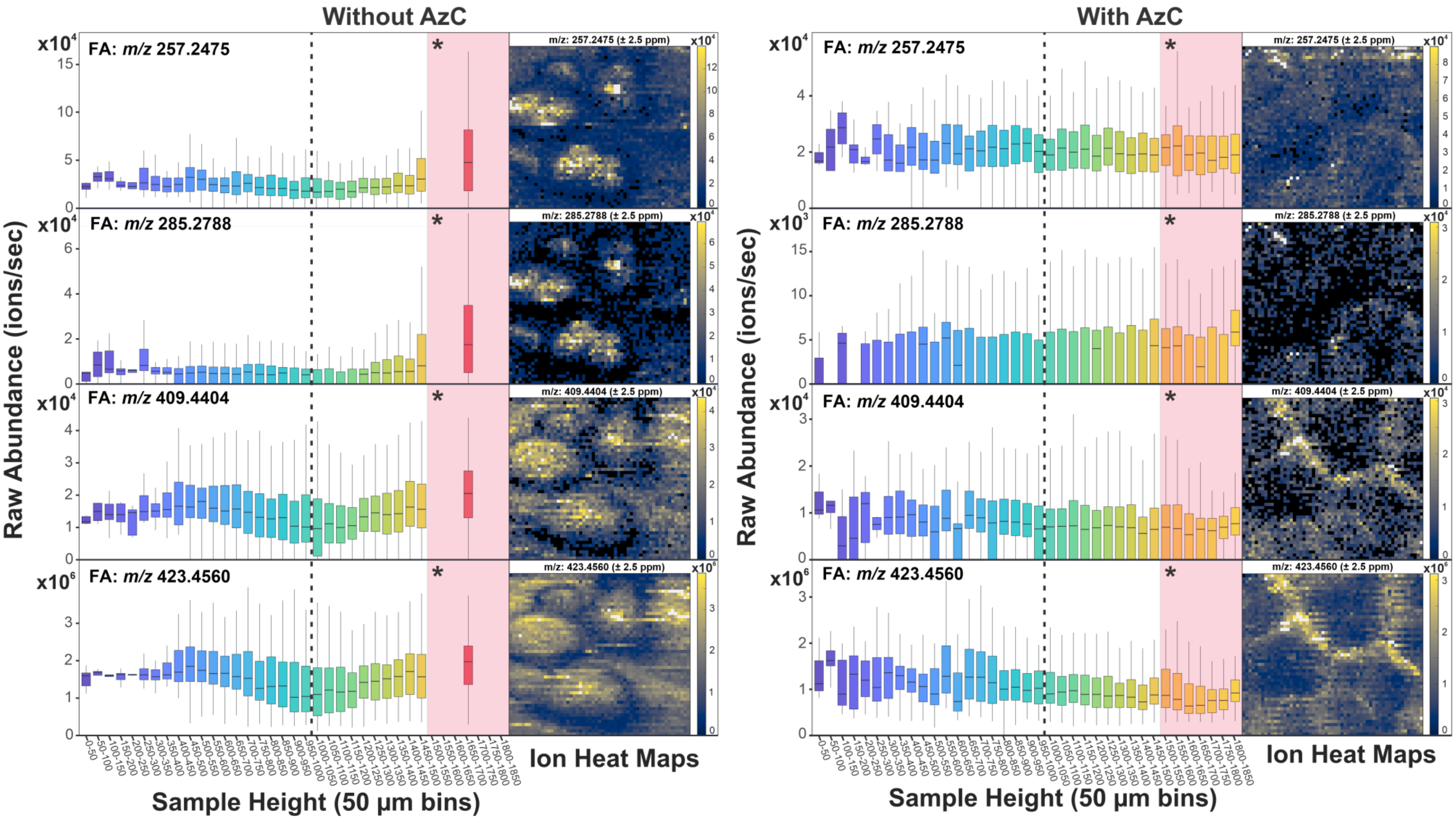

Figure S1. Boxplots of abundance vs. topographic height (left) matched to their corresponding ion images (right) for all fatty acids (FA) interrogated. Data for the section run without AzC are on the left, and data for the section run without AzC are on the right. The height of the focal plane is marked by the dotted line, and the interval of the out-of-range height measurements for the sample run without AzC is marked in red on the boxplots and denoted with an asterisk. All data for out of range measurements in the section run without AzC was compiled into a single box representing the corresponding interval of topographic heights based on prior measurements with calipers.

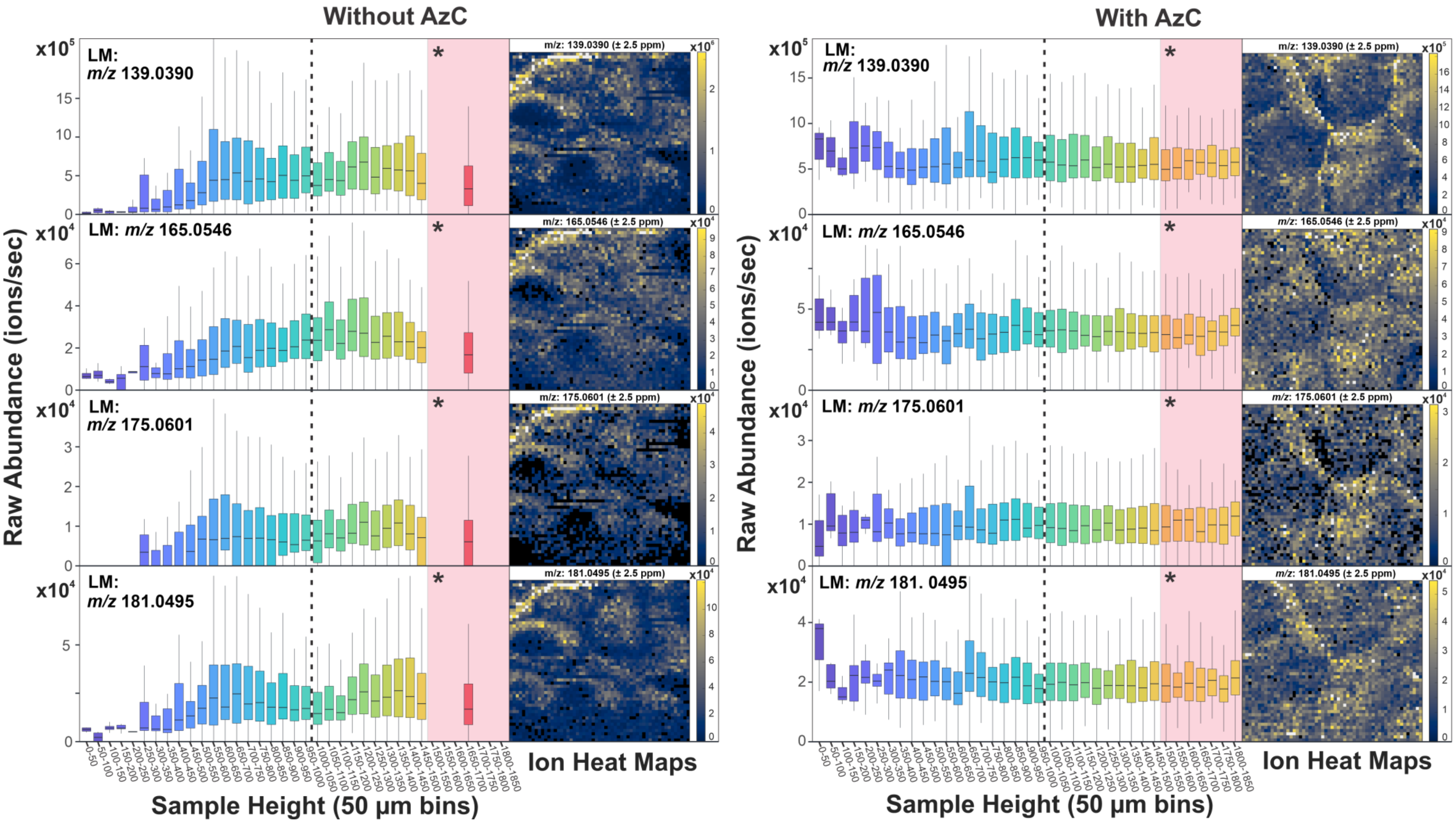

Figure S2. Boxplots of abundance vs. topographic height (left) matched to their corresponding ion images (right) for all lignin-related metabolites (LM) interrogated. Data for the section run without AzC are on the left, and data for the section run without AzC are on the right. The height of the focal plane is marked by the dotted line, and the interval of the out-of-range height measurements for the sample run without AzC is marked in red on the boxplots and denoted with an asterisk. All data for out of range measurements in the section run without AzC was compiled into a single box representing the corresponding interval of topographic heights based on prior measurements with calipers.

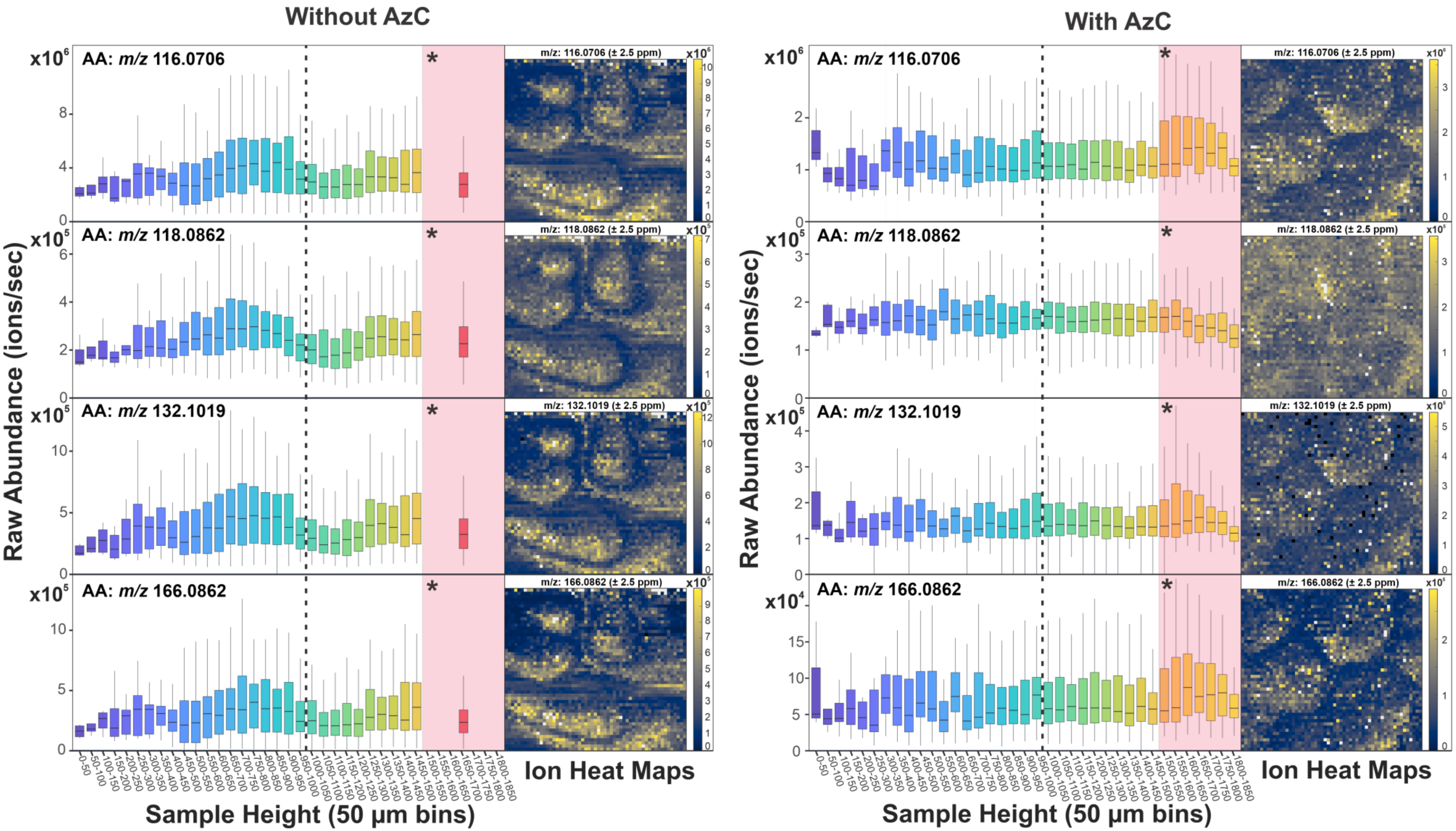

Figure S3. Boxplots of abundance vs. topographic height (left) matched to their corresponding ion images (right) for all amino acids (AA) interrogated. Data for the section run without AzC are on the left, and data for the section run without AzC are on the right. The height of the focal plane is marked by the dotted line, and the interval of the out-of-range height measurements for the sample run without AzC is marked in red on the boxplots and denoted with an asterisk. All data for out of range measurements in the section run without AzC was compiled into a single box representing the corresponding interval of topographic heights based on prior measurements with calipers.

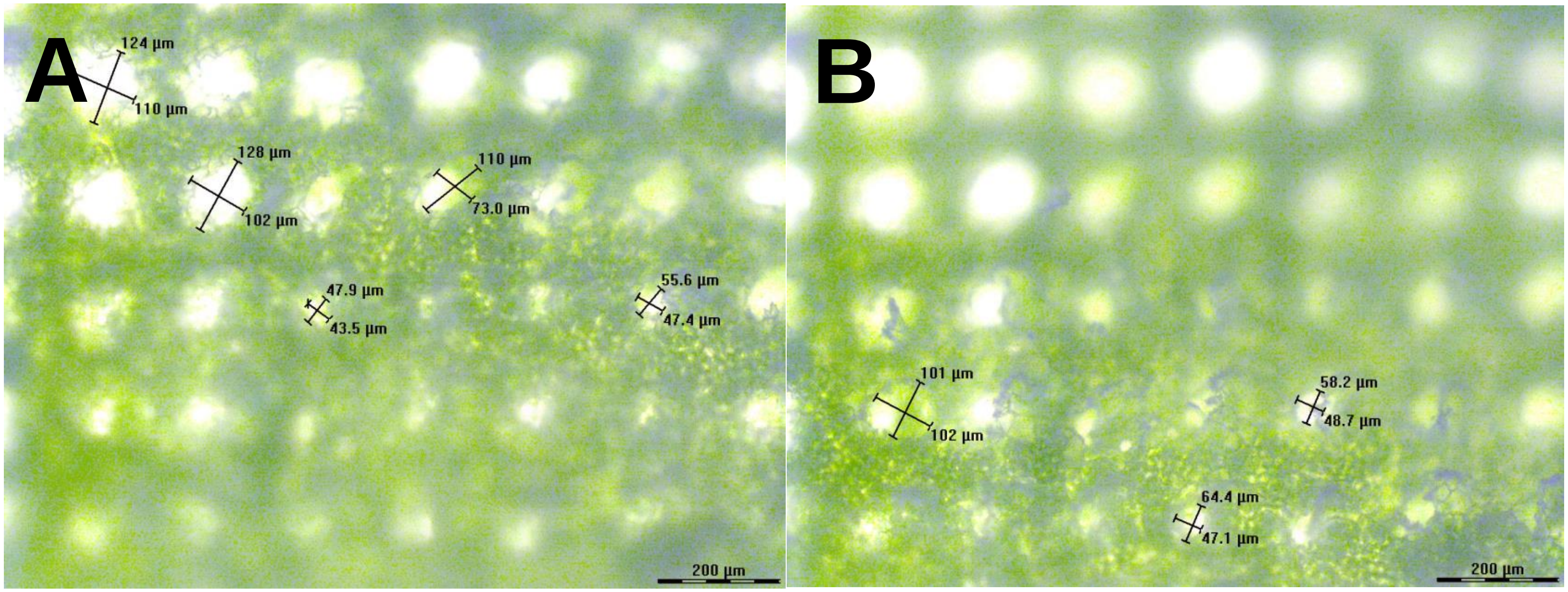

Figure S4. Magnified images of ablation spots without AzC applied. The ragged edges suggest differences in ablation between different layers and are less visible at lower magnification.
